# Stress-Induced Visceral Hypersensitivity in Maternally Separated Rats Can Be Reversed by Peripherally Restricted Histamine-1-Receptor Antagonists

**DOI:** 10.1371/journal.pone.0066884

**Published:** 2013-06-12

**Authors:** Oana I. Stanisor, Sophie A. van Diest, Zhumei Yu, Olaf Welting, Noor Bekkali, Jing Shi, Wouter J. de Jonge, Guy E. Boeckxstaens, Rene M. van den Wijngaard

**Affiliations:** 1 Tytgat Institute for Liver and Intestinal Research, Academic Medical Center, Amsterdam, The Netherlands; 2 Department of Neurobiology, Tongji Medical College, HUST, Wuhan, People’s Republic of China; 3 Division of Gastroenterology, University Hospital Gasthuisberg, Catholic University of Leuven, Leuven, Belgium; Hosptial Infantil Universitario Niño Jesús, CIBEROBN, Spain

## Abstract

**Background:**

The histamine-1 receptor (H1R) antagonist ketotifen increased the threshold of discomfort in hypersensitive IBS patients. The use of peripherally restricted and more selective H1R antagonists may further improve treatment possibilities. We examined the use of fexofenadine and ebastine to reverse post-stress visceral hypersensitivity in maternally separated rats.

**Methods:**

The visceromotor response to colonic distension was assessed in adult maternally separated and nonhandled rats pre- and 24 hours post water avoidance. Subsequently rats were treated with vehicle alone or different dosages of fexofenadine (1.8 and 18 mg/kg) or ebastine (0.1 and 1.0 mg/kg) and re-evaluated. Colonic tissue was collected to assess relative RMCP-2 and occludin expression levels by Western blot and histamine-1 receptor by RT-qPCR. β-hexosaminidase release by RBL-2H3 cells was used to establish possible mast cell stabilizing properties of the antagonists.

**Key results:**

Water avoidance only induced enhanced response to distension in maternally separated rats. This response was reversed by 1.8 and 18 mg/kg fexofenadine. Reversal was also obtained by 1.0 but not 0.1 mg/kg ebastine. RMCP-2 expression levels were comparable in these two ebastine treatment groups but occludin was significantly higher in 1.0 mg/kg treated rats. There were no differences in histamine-1 receptor expression between nonhandled and maternally separated rats. Fexofenadine but not ebastine showed mast cell stabilizing quality.

**Conclusions:**

Our results indicate that the peripherally restricted 2^nd^ generation H1-receptor antagonists fexofenadine and ebastine are capable of reversing post stress visceral hypersensitivity in rat. These data justify future IBS patient trials with these well tolerated compounds.

## Introduction

The functional gastrointestinal disorder irritable bowel syndrome (IBS) is characterized by abdominal pain or discomfort associated with defecation or change in bowel habit.[1] Increased perception to gastrointestinal stimuli, so called visceral hypersensitivity, and barrier dysfunction are considered important pathophysiological mechanisms in IBS. Stress is an important trigger for IBS-symptoms and preclinical investigations suggest that barrier- and sensitivity changes may relate to stress-induced degranulation of intestinal mucosal mast cells.[2]–[4] A recent clinical trial with the mast cell stabilizer and histamine-1-receptor (H1R) antagonist ketotifen confirmed the possible relevance of this cell type.[5] Ketotifen not only decreased abdominal pain and other IBS symptoms but also improved health related quality of life and increased the threshold of discomfort in hypersensitive patients. However, the exact working mechanism of ketotifen remained elusive. Investigations comparing pre- and post-therapy mediator release by submerged rectal biopsies did not support a role for mast cell stabilization. Consequently, it was suggested that H1R antagonism was the main molecular mode of action in this trial.

*Ex vivo* investigations performed by Barbara *et al.* indicated that a mediator present in IBS biopsy-supernatants induced H1R-dependent mesenteric afferent nerve discharge and Ca^2+^-mobilisation in cultured rat DRG neurons.[6] In addition, mucosal biopsies from IBS patients showed a significant increase in H1R mRNA levels over controls.[7] Similar to the ketotifen trial, these results suggested that H1R-targeting may be an attractive treatment option in IBS. However, ketotifen has low H1R selectivity and is known to cross the blood-brain barrier and cause central side effects.[8], [9] Consequently, possibilities to increase therapeutic dose for enhanced effectiveness are limited and evaluation of other, peripherally restricted, H1-receptor antagonists may proof beneficial. In the nineteen eighties second generation non-sedating H1-antihistamines became available and by now this group of antihistamines comprises more than 45 different compounds, including fexofenadine and ebastine.[8] In clinical trials these compounds appeared to be safe, effective and well tolerated and they are now routinely being used in the treatment of allergic rhinitis and urticaria.[10], [11] To establish whether these antagonists hold promise for therapeutical interventions in IBS we evaluated them in the IBS-like rat model of maternal separation. Similar to patients, acute stress induces enhanced sensitivity to colorectal distension in previously separated Long Evans rats.[12] This change in sensitivity was shown to be long lasting, one hour of water avoidance induced enhanced sensitivity for up to one month, and could be reversed by the mast cell stabilizer doxantrazole.[13] In the present study we investigated whether fexofenadine and ebastine were also capable of reversing post stress, mast cell dependent, visceral hypersensitivity in the rat maternal separation model. Our results suggest that peripheral H1Rs may be a safe new target for therapeutical intervention in IBS.

## Materials and Methods

### Ethics statement

All procedures were conducted in accordance with the institutional guidelines and approved by the Animal Ethical Committee of the AMC/University of Amsterdam (reference protocol number 100998).

### Animals and maternal separation (MS) protocol

Long-Evans rats (Harlan, Horst, The Netherlands) were bred and housed at the animal facility of the AMC (Amsterdam Medical Centrum, Amsterdam, The Netherlands). Rats were maintained on a normal 12:12-h dark/light cycle and temperature (20–22°C) and provided with food and water *ad libitum*. Separation was accomplished by placing the dams into another cage in a separate room for 180 minutes per day from postnatal day 2 to 14. During separation, cages were placed on a heating pad (30–34°C) to help pups regulate normal body temperature. Pups were weaned on day 22 and subsequently raised in pairs of two. NH pups were nursed normally.

### Colonic distension protocol and water avoidance (WA)

In IBS patients investigations of visceral sensitivity are performed by colorectal distensions: hypersensitive patients perceive pain during luminal distensions at lower volumes or pressures than normal controls.[14] In our investigations in rat, colonic distensions were performed with a latex balloon (Ultracover 8F, International Medical Products, Zutphen, The Netherlands) at the minimum age of 4 months and carried out as described before.[12], [13], [15] A catheter was placed during short isoflurane anesthesia 20 minutes before distensions with graded volumes of water (1.0, 1.5 and 2.0 mL). Length and diameter of the balloon during maximum volume distension were 18 mm and 15 mm respectively. After each 20 second distension period, water was quickly removed and 80 seconds rest was exercised. At adult age rats were subjected to WA stress during which they were positioned on a pedestal surrounded by water for one hour. Earlier investigations indicated that, in contrast to NH rats, WA induces enhanced sensitivity to colonic distension in MS rats.[12]

### Measurement of the visceromotor response to colonic distension and data analysis

Distension of the colon induces contractions of the abdominal musculature, the so called visceromotor response. Quantification of these contractions by electromyography (EMG) is often used to assess visceral pain responses in rodents. We used radiotelemetric transmitters (Physiotel Implant TA10AE-F20; DSI, St Paul, MN, USA) to record these EMG signals in freely moving rats.[12] In short, the transmitter was positioned in the abdominal cavity and two connected electrodes were placed in abdominal muscles. During distension protocols, animals were placed in a standard macralon cage (exact size of the receiver) that was positioned on top of a receiver (Data Sciences International). The receiver was linked to a Biopac MP100 data acquisition system (Biopac Systems Inc., Santa Barbara, CA, USA) and a personal computer via a raw data analog converter (Data Sciences International). Data were acquired with AcqKnowledge software (Biopac Systems Inc., Santa Barbara, CA, USA) and analyzed as described before. Briefly, each 20-s distension period and its preceding 20-s of baseline recording were extracted from the original raw EMG data file. After correction for movement and breathing, data were rectified and integrated. Absolute data sets were then obtained by subtracting the 20-s baseline recording from the 20-s distension result. Similar to earlier publications the final results are given as normalized data sets, which were calculated from the absolute data by setting the 2 mL value of the first (pre-stress) distension at 100%.[12], [13], [15] Area under the curve (AUC) of relative responses was calculated for individual rats and used to show possible changes in visceromotor response within treatment groups. Relative response data were also used to evaluate possible changes on a per volume basis.

### Design of in vivo pharmacological intervention protocols

Animal experiments were performed while the investigator was blinded to administration of drug or vehicle alone (disclosed after evaluation of all tracings). Directly after measuring baseline sensitivity to distension (10:00 AM, day 0), rats were subjected to WA stress and measured again 24 hours post-WA. Subsequently, rats were treated with intraperitoneal fexofenadine hydrochloride (Tocris Bioscience, Bristol, UK), ebastine (Sigma-Aldrich, Zwijndrecht, The Netherlands) or vehicle alone (10% alcohol). Compounds were administered two times at day 1 (11:00 AM and 05.00 PM) and one time at day 2 (30 minutes before the last distension protocol at 09:30 AM). Cumulative dosages (total of 3 intraperitoneal injections in 24 hour timeframe) were 1.8 mg/kg or 18 mg/kg for fexofenadine and 0.1 mg/kg or 1 mg/kg for ebastine.

### RT-qPCR

To avoid possible distension related effects on H1R expression levels, vehicle treated NH and MS rats were sacrificed 7 days after the last distension protocol. Total RNA was isolated from colonic tissue of NH and MS rats using TRIzol (Invitrogen, Breda, The Netherlands) according to manufacturer’s protocol. Following DNAse treatment, cDNA was obtained by using RevertAid First Strand cDNA Synthesis Kit (Fermentas, Waltham, MA, USA) Quantitative PCR was performed with SYBR Green in the LightCycler480 system (Roche) using a default 60° program. Primer pairs used for H1R were; sense, CTTCTACCTCCCCACTTTGCT, antisense: TTCCCTTTCCCCCTCTTG and for the housekeeping gene Ppib[15]: sense, GCAAGCACGTGGTTTTCGGC, antisense: TGTGAGGGAATCGACAGGACCC.

### Western blotting

In contrast to tissues used for RT-qPCR H1R evaluation, ebastine treated rats were sacrificed directly after the last distension protocol. This tissue was than used to semi quantitatively assess direct effects of ebastine treatment on RMCP-2 and occludin expression levels. Distal colon was dissected, homogenized in lysis buffer (Cell Signaling, Danvers, MA, USA) and assessed by SDS-polyacrylamide gel electrophoresis and Western blotting. Blots were cut at appropriate kD and evaluated for expression of the rat chymase analogue RMCP-2 (polyclonal anti-RMCP-2, Moredun Scientific, Penicuik, Scotland), the tight junction protein occludin (rabbit-anti-occludin, Zymed, San Francisco (CA), USA) and GAPDH (mouse-anti-GAPDH, Millipore, Amsterdam, The Netherlands). Peroxidase-labeled secondary antibody was visualized with Lumi-light plus (Roche Diagnostics, Almere, The Netherlands) and densitometric analyses were carried out with the image processing program ImageJ (http://rsb.info.nih.gov/ij/).

### In vitro mast cell degranulation experiments and beta-hexosaminidase asay

RBL-2H3 cells were used to evaluate the possible mast cell stabilizing effect of fexofenadine and the active metabolite of ebastine; carebastine[11] (Santa Cruz, Heidelberg, Germany). After 30 minutes pre-treatment with these compounds (10 µM, 100 µM or vehicle alone)[16] cells were stimulated with compound C48/80 (Sigma-Aldrich, 100 µg/ml, 500 µg/ml, 1 mg/ml or vehicle alone) for 1 hour. β-hexosaminidase release was quantified by using 4-methylumbelliferyl glucaosaminide as a substrate. Fluorescence was measured at an emmision wavelength of 450 nm and an excitation wavelength of 360 nm. Release of β-hexosaminidase was calculated as a percentage of total cellular content.

### Statistical analysis

Statistical calculations were performed using SPSS for windows (version 11.5.2). VMR data were analysed with the Wilcoxon signed ranks test which was applied for the area under the curve (AUC) of the relative response (normalized data) to colonic distension. Possible statistical differences in Western blot and RT-qPCR evaluations were assessed by Mann-Whitney test.

## Results

### In vivo fexofenadine treatment

**Figure 1:**
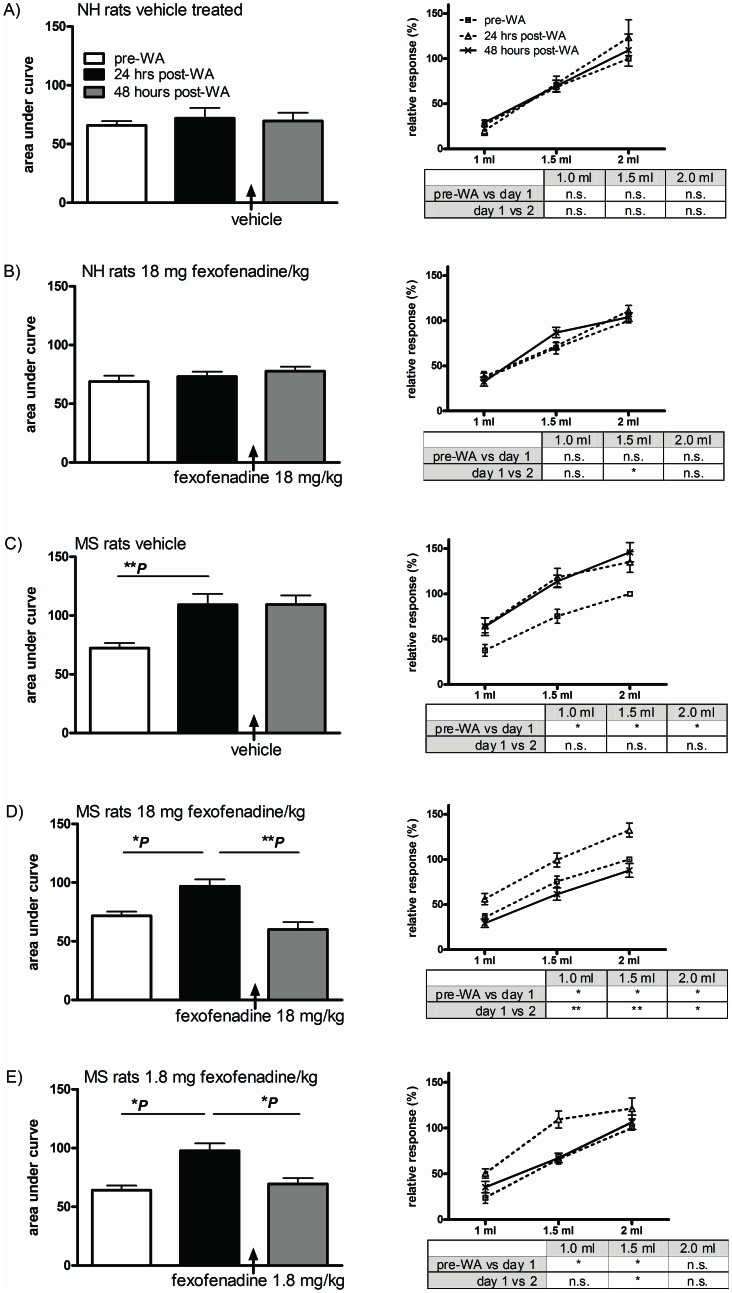
In vivo post stress fexofenadine treatment. The visceromotor response to distension was measured pre-WA and 24 and 48 hours post-WA in NH and MS rats. Fexofenadine or vehicle was administered 3 times between 24- and 48 hours measurements (cumulative dosages 18 and 1.8 mg/kg). Responses to distension are depicted as AUC (left side histograms) and per volume (right side line-diagrams, corresponding statistics in lower right side tables). NH rats did not become hypersensitive to distension and fexofenadine treatment did not change sensitivity levels (figures A and B). In MS rats WA induced enhanced sensitivity to distension in all 3 treatment groups (figures C, D and E). Treatment with 18 and 1.8 mg fexofenadine/kg (figure D and E respectively) but not vehicle alone (C) was able to reverse stress induced visceral hypersensitivity. All data are presented as mean ± SEM, all groups n = 8 or 9 rats, *P<0.05 and **P<0.01.

We established whether a) WA induced post stress hypersensitivity to distension in NH and MS rats and b) whether fexofenadine was capable of reversing sensitivity changes. As published before[12], WA was unable to induce post stress visceral hypersensitivity in NH rats (figures 1A and 1B, white vs black bars) and intraperitoneal post stress administration of high dose fexofenadine (18 mg/kg) did not induce sensitivity changes in these animals (figure 1B, black vs grey bar). In MS rats WA led to increased post-WA AUC in all 3 treatment groups (figure 1C, D and E; *P<0.05, **P<0.01). Enhanced post-stress sensitivity levels were not affected by vehicle treatment alone (figure 1C) but treatment with 18 and 1.8 mg fexofenadine/kg effectively reversed visceral hypersensitivity (figure 1D and 1E respectively).

Per volume comparisons (right side line-diagrams and accompanying statistics-boxes in figure 1A–E) corroborated AUC-data for all MS groups except rats treated with 1.8 mg fexofenadine/kg. In the latter group fexofenadine-induced reversal of hypersensitivity was not significant for 1.0 and 2.0 ml distension volumes. Antagonist treatment did not lead to changes in compliance as assessed by pressure-volume curves (data not shown).

**Figure 2:**
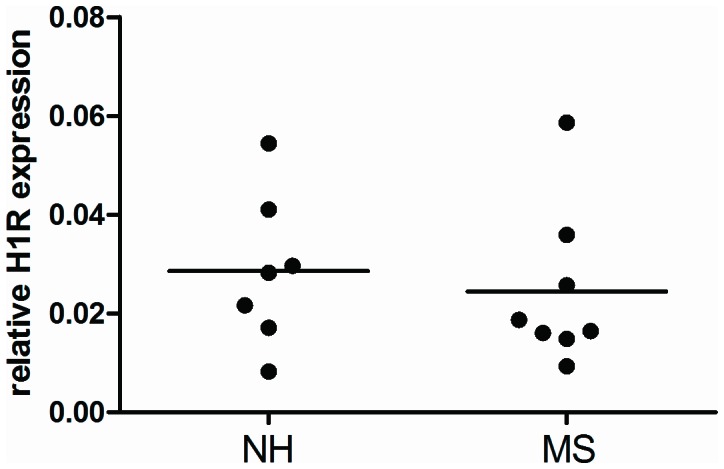
Relative colonic expression values for the *histamine H1 receptor* gene. H1R mRNA expression was evaluated relative to the housekeeping gene *Ppib* in colonic samples of NH and MS rats. Tissue was collected 7 days post vehicle treatment and distensions. There were no significant differences.

H1R gene expression was then determined in colonic tissue of vehicle treated NH and MS rats. Tissue was collected 7 days after the final distension series to avoid protocol induced effects on receptor expression levels. Sufficient yield of RNA was obtained from all but 2 vehicle treated nonhandled rats. As shown in figure 2 there were no significant differences between NH and MS rats.

### In vivo ebastine treatment

**Figure 3:**
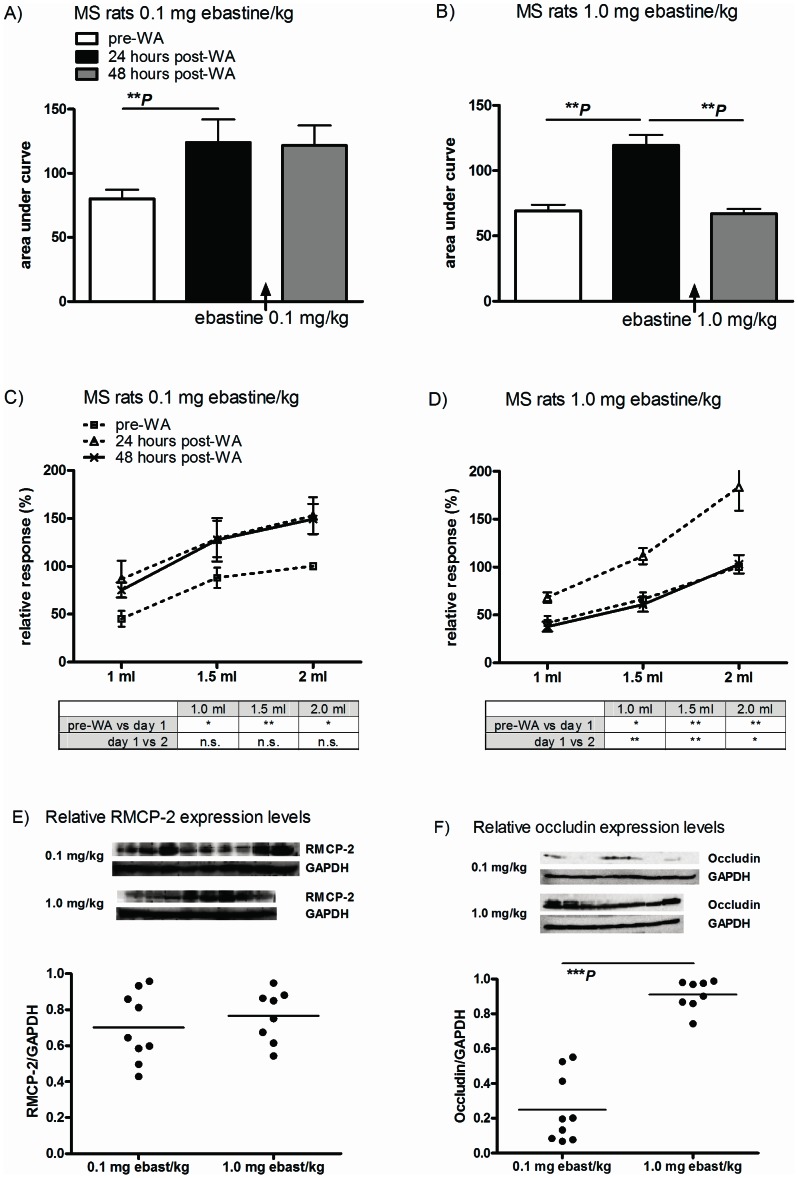
In vivo post stress ebastine treatment. Sensitivity to distension was measured pre-WA and 24 and 48 hours post-WA in MS rats. Ebastine (cumulative dose 0.1 or 1.0 mg/kg) was administered 3 times between 24- and 48 hours measurements (please refer to figure 1C for vehicle treatment group). WA induced increased AUC in both groups (figure A and B, white vs black bars) and a cumulative dose of 1.0 (B) but not 0.1 mg ebastine/kg (A) was able to reverse post-WA hypersensitivity. Line-diagrams of per volume responses show similar results: significantly increased, WA-induced, response to distension for all volumes that were reversed in 1.0 mg/kg but not 0.1 mg/kg treated rats. Semi-quantitative evaluation of (distal) colonic RMCP-2 levels by Western blot showed no difference in expression (E). Compared to 0.1 mg/kg treated rats, occludin expression was significantly higher in 1.0 mg/kg treated rats (F). All data are depicted as mean ± SEM (n = 9 and 8 rats per group). Statistical differences: **P<0.01 and ***P<0.001.

Post WA hypersensitivity to distension did not occur in NH rats and remained unaltered upon fexofenadine treatment. Further, due to their broad clinical use, we know that fexofenadine as well as ebastine are well tolerated in the human setting. Therefore, we choose not to sacrifice additional NH rats to reconfirm results obtained with fexofenadine; ebastine was only evaluated in MS rats. AUC comparisons indicated that WA-induced hypersensitivity to distension could be reversed by an accumulative dose of 1.0 mg ebastine/kg (figure 3B, black vs grey bars, **P<0.01) but not 0.1 mg/kg (figure 3A). Statistical evaluations on a per volume basis (line-diagrams and statistics boxes in figures 3 C and D) showed similar results: we observed a significant post-WA increase for all 3 distension volumes in both treatment groups but these responses could only be reversed in 1.0 mg/kg treated rats.

Rats were sacrificed directly after distensions at the 48 hours time point and selected tissue samples (i.e. tissue not distended by balloon) from distal colon were gathered and evaluated by semi quantitative Western blot. Densitometric analysis of RMCP-2 expression levels (figure 3E) showed no differences between two treatment groups. However, compared to 0.1 mg/kg treated rats, occludin levels were significantly higher in rats treated with 1.0 mg ebastine/kg (figure 3F, ***P<0.001).

### H1R antagonist mediated modulation of C48/80 induced RBL-2H3 degranulation

**Figure 4:**
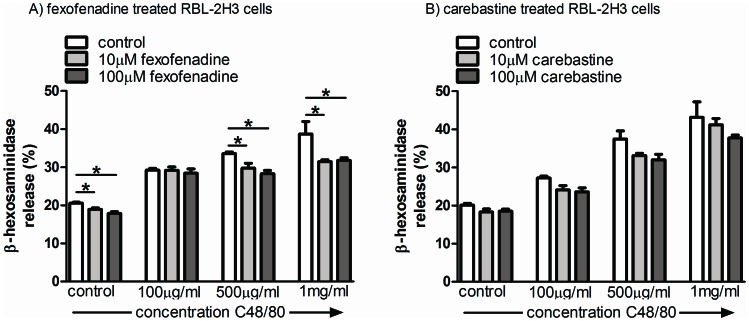
Modulation of Compound 48/80-induced RBL-2H3 degranulation. Compound 48/80 treatment of RBL- 2H3 cells induced a concentration dependent release of β-hexosaminidase. 30 minutes pre-incubation with 10 and 100 µM fexofenadine reduced β-hexosaminidase release in control cells and cells treated with 500 and 1000 µg Compound 48/80/ml (figure A). Carebastine pretreatment did not show an effect on Compound 48/80 stimulated RBL-2H3 cells (figure B). Results are a representative example of 3 independent experiments and expressed as mean ± SEM (4 wells per condition, *P<0.05).

We monitored C48/80-induced release of β-hexosaminidase by RBL-2H3 cells to demonstrate possible mast cell stabilizing qualities of fexofenadine and ebastine. C48/80 induced a dose dependent release of the enzyme from RBL-2H3 cells (figures 4A and B). 30 minutes fexofenadine pre-treatment reduced baseline release as well as release induced by 500 and 1000 µM C48/80 (figure 4A, *P<0.05). In contrast, the ebastine metabolite carebastine was unable to prevent degranulation in any of the C48/80 concentrations tested (figure 4B).

## Discussion

In a recent clinical trial the use of ketotifen was shown to improve health related quality of life, increase threshold of discomfort and decrease IBS symptoms in hypersensitive IBS patients. Ex vivo evaluations of pre- and post therapy rectal biopsies suggested that positive trial outcome was not due to the mast cell stabilizing effect of ketotifen but may be related to the H1R-antagonistic properties of this compound. Because ketotifen treatment may be associated with central side effects we evaluated, in the rat MS-model, the potential use of peripherally restricted H1R-antagonists. Our data show that the selective antagonists fexofenadine and ebastine are both capable of reversing post-WA visceral hypersensitivity.

Early life stressors are known to predispose for IBS at adult age[17], [18] and MS in rat is a well accepted animal model to mimic such predisposing factors.[2] Although in some rat strains neonatal MS as such is enough to induce an IBS-like phenotype, Long Evans rats have to be subjected to an additional acute stress (e.g. WA) at adult age to bring out the hypersensitive phenotype.[12] This is similar to what is observed in IBS patients were acute stress is a known trigger for visceral hypersensitivity. In MS Long Evans rats we were able to show that mast cell degranulation plays a pivotal role in the development of stress-induced visceral hypersensitivity and loss of barrier integrity. Pre stress treatment with the mast cell stabilizer doxantrazole prevented and post-stress treatment reversed WA-induced hypersensitivity to distension and occludin degradation.[13], [15] Although histamine may be one of the mediators released upon mast cell activation direct evidence for a role in MS associated visceral hypersensitivity was not available so far. Histamine is however one of the mast cell mediators implicated in the activation of afferent-expressed TRPV1[19] and post stress treatment with the selective TRPV-1 antagonist SB-705498 reversed visceral hypersensitivity in the MS model.[15] Thus, we considered MS a suitable model to evaluate the possible use of H1R antagonists in the treatment of stress-induced IBS-like phenotypical changes. Importantly, because a possible future treatment protocol would aim to reverse complaints in IBS patients we evaluated these compounds in a post stress treatment protocol. Fexofenadine as well as ebastine were capable of reversing post-WA visceral hypersensitivity.

The outcome of these experiments contradicts earlier investigations involving the in vivo use of the calcium ionophore BrX-537A (Bromolasolacid). Coelho *et al.* showed that intraperitoneal administration of BrX-537A led to mast cell degranulation and enhanced sensitivity to colorectal distension.[20] The observed visceral hypersensitivity was prevented by 5-HT_1a_ receptor antagonist but not by histamine receptor-1, -2 and -3 antagonists. In these experiments only one dosage of H1R antagonist (1 mg chlorophenizamine/kg) was used and we can not rule out the possibility that it was to low to be effective. Alternatively, histamine release may occur in both experimental conditions but release only has consequences relevant to visceral sensitivity when rats are predisposed to react to this mediator (e.g. by increased H1R expression). In relation to this, mucosal biopsies of IBS patients were indeed shown to have increased H1R mRNA over controls.[7] Therefore, we investigated the possibility of enhanced post-WA H1R expression in MS rats but expression was not increased over NH rats. Although the same approach to H1R evaluation was successfully used by Sander *et al.* [7], we can not exclude the possibility that existing differences between groups were diluted out because we evaluated whole tissue specimens instead of isolated sensory neurons. Another explanation for the observed discrepancy with the earlier BrX-537A investigations may be found in an often neglected aspect of in vivo visceral sensitivity investigations. The calcium ionophore study evaluated prevention of mast cell induced hypersensitivity whereas our H1R antagonist data describe reversal of mast cell dependent hypersensitivity. The difference is not ‘just semantics’. Recent data on the use of α-helical-CRF (9–41) showed that pre-WA targeting of CRF receptors prevented, but post-WA targeting could not reverse stress induced visceral hypersensitivity.[13] Similarly, histamine may play a role in prolonged mast cell dependent hypersensitivity but not during an acute phase such as investigated in the BrX-537A experiments.

Because ketotifen that was used in the IBS clinical trial has H1R antagonistic as well as mast cell stabilizing qualities we also evaluated fexofenadine and carebastine (the active metabolite of ebastine) for possible mast cell stabilizing effects. Data obtained with RBL-2H3 cells indicated that fexofenadine had some weak mast cell stabilizing quality and carebastine, although results did not reach significance, showed the same trend. However, the level of stabilization was far from complete and can never explain the successful in vivo reversal of post stress visceral hypersensitivity by these compounds. Further, our results on RMCP-2 tissue expression levels suggest that in vivo mast cell degranulation is not altered by their use. In an earlier study we showed that in vivo post stress mast cell degranulation is associated with a decrease in tissue RMCP-2 levels.[13] In the current investigations high (1.0 mg/kg) but not low dose (0.1 mg/kg) ebastine effectively reversed visceral hypersensitivity whereas semi-quantitative evaluation of corresponding colonic tissues did not show differences in RMCP-2 expression levels. The latter data suggest equal level of mast cell degranulation in treatment groups and confirmed the lack of in vitro stabilization by ebastine. Therefore, at least for ebastine, we suggest that H1R antagonism rather than mast cell stabilization was the in vivo mechanism of action.

In addition to RMCP-2, homogenized colonic tissue samples of ebastine-treated rats were evaluated for occludin expression. In a previous study we observed post-stress degradation of this tight junction protein in stripped colonic mucosa of MS Long Evans rats.[13] Here, ebastine-induced reversal of visceral hypersensitivity was associated with high- and failure to reverse with low- level occludin expression. How this change is relevant for the observed visceral hypersensitivity is not clear yet. However, barrier dysfunction is thought to be an important pathophysiological mechanism in IBS and in patient biopsies occludin degradation was shown to correlate with abdominal pain intensity scores.[21], [22] The latter may be explained by enhanced mucosal influx of luminal antigens and/or bacteria leading to subsequent immune cell and afferent activation.[4] In relation to this, *in vivo* occludin depletion by selective siRNA-induced knock down in mouse intestine was indeed shown to enhance macromolecular flux across intestinal epithelial cells.[23] A direct link between barrier dysfunction and hypersensitivity to distension was shown in rats where intra-colonic infusion of a tight junction blocker prevented stress induced rectal hypersensitivity.[24] In the present investigations we only obtained a limited dataset on occludin expression. Future investigations should aim to establish whether ebastine treatment, next to possible effects on afferent expressed H1R[6], can also lead to antagonist mediated restoration of barrier function.

At present peripherally restricted H1R-selective antihistamines are the most broadly used medications in the treatment of allergic diseases. In consequence, compounds like ebastine and fexofenadine have been extensively investigated regarding clinical pharmacology and safety. The present study indicates that these compounds are capable of reversing post stress visceral hypersensitivity. Since this trait may be relevant to IBS we suggest that peripheral H1Rs can be a safe new target for IBS therapy.
